# Safety and tracking of intrathecal allogeneic mesenchymal stem cell transplantation in healthy and diseased horses

**DOI:** 10.1186/s13287-018-0849-6

**Published:** 2018-04-10

**Authors:** Danielle Jaqueta Barberini, Monica Aleman, Fabio Aristizabal, Mathieu Spriet, Kaitlin C. Clark, Naomi J. Walker, Larry D. Galuppo, Rogério Martins Amorim, Kevin D. Woolard, Dori L. Borjesson

**Affiliations:** 1 Veterinary Institute for Regenerative Cures and the Department of Pathology, Microbiology & Immunology, University of California, Davis, USA; 2 Department of Medicine & Epidemiology, University of California, Davis, USA; 3 Department of Surgical & Radiological Sciences, University of California, Davis, USA; 4 Department of Veterinary Clinics, São Paulo State University “Julio de Mesquita Filho” – UNESP, Botucatu, SP Brazil

**Keywords:** Mesenchymal stem cells, Intrathecal, Neurology, Scintigraphy, Adipose tissue, Cerebrospinal fluid

## Abstract

**Background:**

It is currently unknown if the intrathecal administration of a high dose of allogeneic mesenchymal stem cells (MSCs) is safe, how MSCs migrate throughout the vertebral canal after intrathecal administration, and whether MSCs are able to home to a site of injury. The aims of the study were: 1) to evaluate the safety of intrathecal injection of 100 million allogeneic adipose-derived MSCs (ASCs); 2) to assess the distribution of ASCs after atlanto-occipital (AO) and lumbosacral (LS) injection in healthy horses; and 3) to determine if ASCs homed to the site of injury in neurologically diseased horses.

**Methods:**

Six healthy horses received 100 × 10^6^ allogeneic ASCs via AO (*n* = 3) or LS injection (*n* = 3). For two of these horses, ASCs were radiolabeled with technetium and injected AO (*n* = 1) or LS (*n* = 1). Neurological examinations were performed daily, and blood and cerebrospinal fluid (CSF) were evaluated prior to and at 30 days after injection. Scintigraphic images were obtained immediately postinjection and at 30 mins, 1 h, 5 h, and 24 h after injection. Three horses with cervical vertebral compressive myelopathy (CVCM) received 100 × 10^6^ allogeneic ASCs labeled with green fluorescent protein (GFP) via AO injection and were euthanized 1–2 weeks after injection for a full nervous system necropsy. CSF parameters were compared using a paired student’s *t* test.

**Results:**

There were no significant alterations in blood, CSF, or neurological examinations at any point after either AO or LS ASC injections into healthy horses. The radioactive signal could be identified all the way to the lumbar area after AO ASC injection. After LS injection, the signal extended caudally but only a minimal radioactive signal extended further cranially. GFP-labeled ASCs were not present at the site of disease at either 1 or 2 weeks following intrathecal administration.

**Conclusions:**

The intrathecal injection of allogeneic ASCs was safe and easy to perform in horses. The AO administration of ASCs resulted in better distribution within the entire subarachnoid space in healthy horses. ASCs could not be found after 7 or 15 days of injection at the site of injury in horses with CVCM.

**Electronic supplementary material:**

The online version of this article (10.1186/s13287-018-0849-6) contains supplementary material, which is available to authorized users.

## Background

Mesenchymal stem cells (MSCs) have been evaluated as a potential treatment for a variety of diseases, including neurological [1, 2], musculoskeletal [3–5], autoimmune [6–8], and inflammatory [9] disorders, in many species.

The central nervous system (CNS) has a limited capacity for regeneration making stem-cell based therapy a promising alternative due to its immunomodulatory, anti-inflammatory, neuroprotective, antiapoptotic, and proneurogenic characteristics [10–12].

Several neurological disorders in humans have raised particular interest for stem cell-based therapy, including multiple sclerosis, spinal cord injury, Parkinson’s disease, stroke, Huntington’s disease, amyotrophic lateral sclerosis, and Alzheimer’s disease, which have been studied in experimental models and clinical trials [13–19]. In equine medicine, neurological disorders affecting the brain and spinal cord can present a therapeutic challenge [20–23] and many horses with neurological diseases such as equine protozoal myeloencephalitis (EPM) and cervical vertebral compressive myelopathy (CVCM or ‘Wobbler’s disease’) can have neurological sequelae even after the recommended treatment.

An important first step when considering a cell type for use in regenerative medicine is to investigate the ability of that cell to migrate, engraft, and survive at sites of injury without causing significant adverse side effects.

Preliminary studies have demonstrated the safety of intrathecal MSC administration in rats [24], humans [25], dogs [26], rabbits [27], and horses [28]. MSCs could potentially be administered intrathecally to horses at three different sites: the atlanto-occipital (AO) cisterna [29], the intervertebral space between the first and second cervical vertebrae (C1–C2) [30], and the lumbosacral (LS) space [29]. Site selection could depend on neuroanatomical localization of lesions (the administration site preferred closest to the lesion), the clinician’s expertise, patient cooperation, and pharmacological protocol (e.g., sedation versus general anesthesia) [31]. It is currently unknown whether MSCs administered intrathecally would be able to migrate throughout the subarachnoid space and home to a diseased site. Developing protocols to administer allogeneic MSCs would permit immediate cell therapy in acute and subacute neurological diseases and would eliminate variation in ex vivo expansion that can hinder autologous cell use, especially in older animals and humans [32].

The objectives of this study were: 1) to determine the safety of a relatively high dose of intrathecal adipose-derived MSCs (ASCs) in healthy horses; 2) to track ASCs after AO and LS administration in healthy horses; and 3) to determine if ASCs would migrate to the diseased spinal cord site in horses with severe neurologic disease and survive in the central nervous system for 2 weeks following inoculation.

We hypothesized that the intrathecal administration of allogeneic ASCs into healthy horses would be safe, as measured by clinical neurological evaluation, blood work, and cerebrospinal fluid (CSF) analysis, that the ASCs would distribute throughout the subarachnoid space after both LS and AO injection, and that ASCs administered in horses with severe neurological disease would migrate to the neurological injury site and would survive for 2 weeks.

## Methods

### Animal selection

For healthy horses, six clinically healthy adult mares of Thoroughbred (*n* = 2), Quarter Horse (*n* = 2), and Warmblood (*n* = 2) breeds from the Center of Equine Health (CEH), UC Davis, USA, were selected for the study. The ages of the mares ranged from 6 to 21 years (mean 12 years). All mares were determined to be healthy based on a normal physical and neurological examination, and by a complete cell blood count and serum biochemical profile.

For diseased horses, three client-owned horses who were going to be euthanized (per the owner’s request) due to moderate to severe neurological signs were donated to the CEH for this study. These horses were donated with presumed CVCM. These horses had a normal physical examination. The neurological examination revealed signs consistent with a symmetrical cervical (C1 to C6) myelopathy. Their neurological deficits were graded as 3 according to a published grading scale [31]. The protocol was approved by UC Davis Institutional Animal Care and Use Committee (IACUC) on 24 June 2015 (protocol no. 18801).

### Study design

This was a non-blinded, randomized study.

For healthy horses, three horses were randomly assigned to receive ASCs via AO injection and three horses to receive ASCs via LS injection. One horse from each group received ASCs radiolabeled with ^99m^technetium-hexamethyl-propylene-amine-oxyme (^99m^Tc-HMPAO; AnazaoHealth, Tampa, FL, USA) for cell tracking studies. A control AO injection of ^99m^Tc-HMPAO without ASCs was performed in one horse. Blood (jugular venipuncture) and CSF were collected prior to and after ASC injection (day 0 and day 30, end of study). Complete neurological clinical examinations were performed prior to ASC injection and once a day for 30 days (the end of the study).

For neurologically diseased horses, three horses with CVCM received ASCs labeled with noninfectious viral vectors transduced with green fluorescent protein (GFP) via AO injection. Blood and CSF were collected prior to and 7 (*n* = 1) or 15 (*n* = 2) days after ASC injection. Additionally, neurological examinations were performed until the end of the study (7 or 15 days). Horses were euthanized at 7 or 15 days postinjection and submitted for a full nervous system necropsy by a board-certified veterinary pathologist.

### CSF collection and analysis

CSF was collected per routine protocol utilizing published anatomical landmarks from the AO or LS space prior to ASC injection and at 30 days postinjection. The CSF was submitted for complete cytological analysis at the William R. Pritchard Veterinary Medical Teaching Hospital (VMTH), UC Davis. Due to the observed and reported seroprevalence in our area [33], an immunofluorescent antibody test (IFAT) for the detection of antibodies against *Sarcocystis neurona* and *Neospora hughesi*, the causative agents of EPM, was preformed concurrently on serum and CSF. If antibodies were present in serum and/or CSF, serum to CSF IFAT antibody titer ratios were calculated to further determine the likelihood of EPM infection in horses with neurologic disease [34].

### Mesenchymal stem cells (MSCs)

ASCs (passage (P)2–P4) were used for injections into healthy and neurologically diseased horses. These cells were obtained from the UC Davis VMTH Regenerative Medicine Laboratory (RML). These samples were originally submitted for MSC expansion for autologous patient treatment. Excess cells not used for treatment were donated for research purposes with written consent of the owner. Cryopreserved MSCs were thawed, washed, and expanded in culture exactly as previously described [35]. Equine MSCs were tested for purity and identity using CD44 (clone CVS18; AbD Serotec, Raleigh, NC, USA), CD29 (clone 4B4LDC9LDH8; Beckman Coulter, Brea, CA, USA), F6B (white blood cell label; gift of Dr. Jeffrey Stott, UC Davis), CD90 (clone DH24A; VMRD, Pullman, WA, USA), MHC I (clone CVS22; AbD Serotec), and MHC II (clone CVS20; Bio-Rad Laboratories, Hercules, CA, USA) (Additional file 1). Prior to administration they were enumerated and confirmed to be viable (trypan blue), sterile (bacterial culture), and negative for endotoxin (PYROGENT Plus LAL Gel Clot Assay; Lonza, Walkersville, MD, USA) and mycoplasma (MycoScope PCR Kit; Genlantis, San Diego, CA, USA).

### GFP-transduction method

The eGFP/luciferase lentivirus (pCCLc-MNDU3-LUC-PGK-EGFP-WPRE) [36] was a gift from Dr. Jan Nolta. Equine MSCs were transduced as previously described for human MSCs [36]. Briefly, equine MSCs were thawed for at least 3 days prior to transduction. Cells were trypsinized, pelleted, resuspended in transduction media (Dulbecco’s modified Eagle’s medium (DMEM), 10% fetal bovine serum (FBS), and 10 μg/mL protamine sulfate) and plated in a T75 flask. Lentivirus was added to the flask (multiplicity of infection (MOI) = ~ 10) and the cells were incubated overnight. After 24 h, 2 volumes of standard media were added to the flask. Cells were passed and counted 3 days after transduction and again 5 days later. eGFP efficiency was determined by fluorescent microscopy and flow cytometry at both time points. Cells were then frozen and/or expanded for injection.

### Radiolabeling with ^99m^Tc-HMPAO

ASCs were labeled with 20 mCi of ^99m^Tc-HMPAO as previously described [37, 38] with minor modifications as follows. ASCs were resuspended in a small volume of media with 10% FBS (∼ 0.5 mL). HMPAO (reconstituted in saline) was added to the ^99m^Tc and incubated for 5 min. Cells were added to make a final concentration of 125 μg/mL HMPAO, 20 mCi^99m^ Tc for cell loading (22 mins). After cell loading, ASCs were washed twice with saline (first wash contained 5% autologous serum), and resuspended in sterile saline. Labeled cells were injected at a concentration of 100 million cells in 5 mL. An aliquot of cells was used to determine viability and label persistence. Labeling efficiency and label persistence at 6 h were assessed as previously described [37, 38]. Cell viability at 6 h was assessed on a sample of ASCs using the trypan blue exclusion test. For the control injection (no ASCs), HMPAO was combined with ^99m^Tc (125μg HMPAO + 10 mCi Tc for 5 min) and then 5 mCi ^99m^Tc-HMPAO was diluted to 5 mL with saline for injection.

### Intrathecal ASC injection

For healthy horses, 100 × 10^6^ ASCs were injected in 5 mL of sterile saline either AO or LS (depending on the group, *n* = 3 each) immediately following CSF collection. One horse in each group received Tc-labeled ASCs and one horse was injected AO with free label (Tc-HMPAO without ASCs; control) after the study was completed.

For neurologically diseased horses (*n* = 3), 100 × 10^6^ ASCs transfected with a retrovirus GFP-bioluminescence construct were injected in 5 mL of sterile saline via AO tap immediately following CSF collection.

### Tracking of ASCs

#### Short-term tracking in healthy horses

##### Scintigraphic imaging

Images were acquired using a gamma camera (IS2 medical Systems, Ottawa, Canada) with a low-energy all-purpose collimator set at 140 keV photoelectric peak and 20% symmetrical window. Lateral images of the spine from the head to the sacrum were obtained using a 1-min static acquisition immediately postinjection and at 30 min, 1 h, 5 h, and 24 h after injection. Small radioactive markers were placed as landmarks for scintigraphic acquisition. Radiographs (Eklin EDR-6, Sound-Eklin, Carlsbad, CA, USA) of the vertebral column from the head to the sacrum were obtained the day after scintigraphy with radiopaque markers in a similar location to the radioactive markers.

##### Scintigraphic interpretation

The presence and distribution of the radioactive signal was assessed subjectively by a board-certified radiologist using the radioactive markers for anatomical localization based on correlation with the radiographs. The persistence at the injection site was quantified over time. A region of interest (ROI) was drawn using the free-hand ROI tool of the DICOM viewer software (Osirix Foundation, Geneva, Switzerland). Persistence was defined as the ratio of detected counts corrected for decay in the ROI at each time point divided by the number of counts initially present in the ROI.

ASCs were transfected with a retrovirus GFP-bioluminescence construct prior to injection exactly as described previously [39]. Equine MSCs are readily transfected with this viral vector [40]. Intrathecal injection was performed in horses under general anesthesia and 100 × 10^6^ labeled ASCs suspended in saline solution was injected into the AO space.

#### Tracking in neurologically diseased horses

##### Necropsy and sample collection

A full nervous system necropsy was performed 15 days (*n* = 2) and 7 days (*n* = 1) after ASC injection. Tissue sections were collected and fixed in 4% paraformaldehyde (PFA) for 3 days (PFA changed daily). Following fixation, samples were cryoprotected in 30% sucrose and then sectioned for routine hematoxylin and eosin (H&E) tissue processing and frozen sections. In brief, each spinal cord segment was sampled for two H&E cross-sections and three frozen cross-sections in an alternating pattern (frozen–H&E–frozen–H&E–frozen), followed by an approximate 1-cm longitudinal section for frozen. Approximately 150 frozen sections were evaluated for each horse. Routine H&E sections were processed for regular histology and stained on an autostainer (Leica Biosystems Inc., IL, USA). Frozen sections were cut on a cryotome (Life technologies, Carlsbad, CA, USA) at 10 μm thickness and stained with 4,6-diamidino-2-phenylindole (DAPI) at 2 μg/mL for 5 min at room temperature prior to mounting. Sections of the nervous system (brain and spinal cord) were imaged on an Evos FL inverted epifluorescence scope (Life technologies, Carlsbad, CA, USA) to detect bioluminescent ASCs.

To assess for the presence of occult GFP-labeled cells, we performed additional immunofluorescence assays using a rabbit-anti-GFP primary antibody (clone D5.1; Cell Signaling, Danvers, MA, USA), visualized using an Alexa594-conjugated goat-anti-rabbit IgG secondary antibody (Leica Biosystems Inc.). Sections from three horses were imaged on both the GFP and 594 (red) channels.

### Anti-ASC alloantibody flow cytometric crossmatch assay

To determine if healthy horses developed any anti-MSC alloantibodies after intrathecal administration of allogenic ASCs, we performed a crossmatch assay exactly as previously described [41]. In brief, cryopreserved ASCs were thawed, washed, incubated in blocking solution, washed, and incubated with horse sera (ASC recipient serum). After incubation, ASCs were washed and incubated with the secondary antibody (rabbit polyclonal antibody to equine IgG-FITC; Abcam, Cambridge, MA, USA). Cells were washed, resuspended in flow buffer, and analyzed on a flow cytometer (Cytomics FC 500; Beckman Coulter). Flow cytometry data were analyzed using FlowJo flow cytometry software (Tree Star Inc., Ashland, OR, USA). Each recipient horse sera was incubated with the ASCs that they received as well as ASCs that they did not receive (irrelevant ASCs) to determine binding specificity. Pre- and post-sera were available for four of the recipient horses.

### Detection of anti-BSA antibodies

An enzyme-linked immunosorbent assay (ELISA) was adapted to detect antibodies directed against the primary bovine protein in FBS, bovine serum albumin (BSA) [42]. This ELISA was performed as previously described to determine if the intrathecal administration of ASCs cultured in FBS resulted in increased anti-BSA titers in healthy horses [41]. Serum samples collected prior to ASC administration and at 30 days and 14 months following ASC administration were available from four of the horses. The fold-increase in color relative to the negative control was determined for each sample.

### Statistical analysis

CSF parameters prior to and after ASC injection were compared using a paired student’s *t* test. A *P* value < 0.05 was considered significant for all analyses.

## Results

### ASC injection into healthy horses

#### High-dose intrathecal ASC injection is safe and well tolerated

ASC administration was readily performed in both standing (LS) and laterally recumbent anesthetized horses (AO). No adverse events were noted during or after ASC administration. The complete blood count and biochemical profile showed no alterations prior to or after ASC injection (with or without ^99m^Tc-HMPAO) for either AO or LS groups. All mares had normal physical and neurological examinations prior and after the administration of ASCs, including the 30 day recheck examination post-injection.

#### High-dose intrathecal administration of ASCs does not alter CSF parameters

There were no statistical differences between AO and LS CSF parameters prior to or after ASC administration; as such, the groups were combined for further analysis. In addition, there were no significant differences in CSF total protein, nucleated cell count, or cell differential prior to or after ASC administration (*P* > 0.05). The majority of horses had normal cell counts, total protein, and cell differential both prior to and after ASC administration. Prior to ASC administration, 2/6 horses (33%) had increased nucleated cell count (9 and 13 cells/μL, reference range ≤ 5 cells/μL) and all horses had a normal total protein concentration (reference range < 100 mg/dL). These changes were not considered clinically relevant in light of normal neurological and physical examination. Thirty days after ASC administration, 1/6 horses (17%) had an increased CSF protein (121 mg/dL) and 5/6 horses (83%) had an increased nucleated cell count (6, 6, 7, 13, and 26 cells/μl).

There were no large or atypical cells (potential MSCs) noted on highly concentrated CSF fluid submitted for cytological review. Antibody titers on IFAT for both blood and CSF were negative for *S. neurona* and *N. hughesi*. Therefore, antibody titer ratios were not calculated.

Data from CSF analyses prior to and after ASC administration are detailed in Table 1.

**Table 1:** Summary of cerebrospinal fluid analytes prior to (pre) and after (post) intrathecal adipose-derived mesenchymal stem cell (ASC) administration in healthy horses

Site of collection	Total protein (mg/dL)		Nucleated cell count (cells/μL)		Neutrophil (%)		Small mono (%)		Large mono (%)	
	Pre	Post	Pre	Post	Pre	Post	Pre	Post	Pre	Post
LS	84	72	13	7	0	10	59	89	41	1
LS	59	67	9	13	2	0	88	91	10	9
LS*	64	64	1	4	19	84	23	15	58	1
AO	88	121	1	6	47	5	39	83	14	12
AO	55	81	< 1	6	0	0	0	96	0	4
AO*	77	85	< 1	26	0	1	0	90	0	2
Mean	71.2	81.7	4.2	10.3	11.3	16.7	34.8	77.3	20.5	4.8
P value	0.18		0.21		0.72		0.06		0.20	

Reference intervals: total protein < 100 mg/dL; nucleated cell count < 5 cells/μL * Technetium-labeled cells

*LS*, lumbosacral; *AO*, atlanto-occipital; *mono*, mononuclear cells

#### ASCs administered AO distributed caudally throughout the vertebral canal whereas ASCs administered LS failed to distribute cranially

**Fig. 1:**
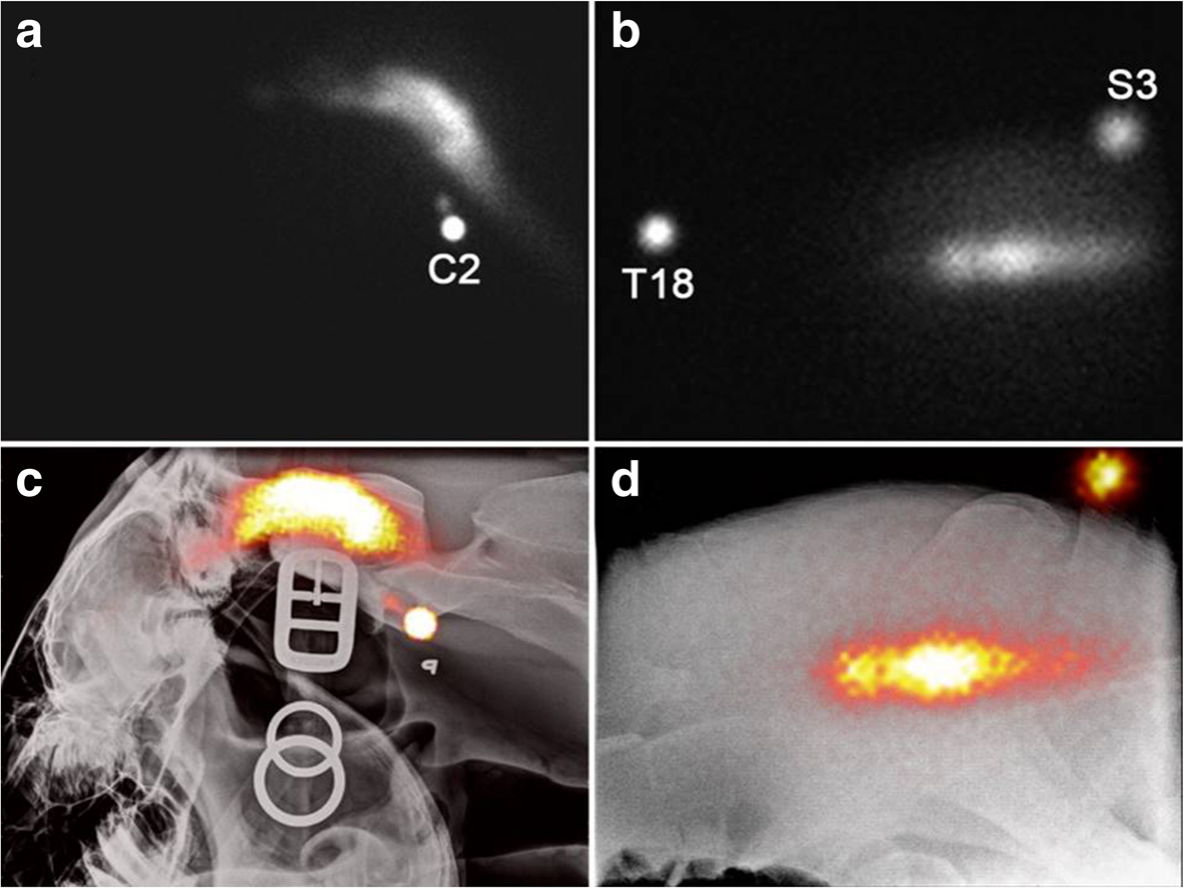
Scintigraphic images obtained immediately after AO (**a**) and LS (**b**) injection of radiolabeled ASCs. The focal round area of intense radioactive signal are external markers placed on the second cervical vertebra (C2), the eighteenth thoracic vertebra (T18), and the third sacral vertebra (S3). The bottom row shows fusion of the scintigraphic images with radiographs after AO (**c**) and LS (**d**) injection to help with anatomical localization of the ASCs

**Fig. 2:**
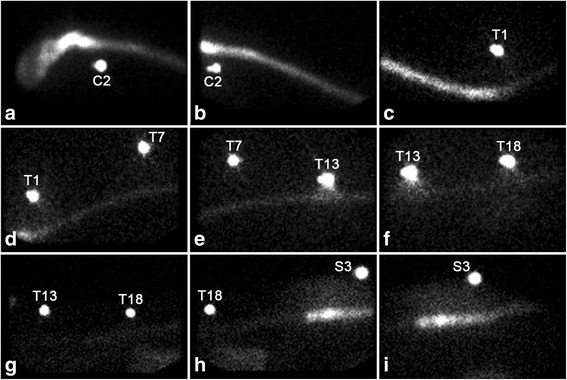
Scintigraphic images obtained 5 h after AO (**a–f**) and LS (**g–i**) injection of radiolabeled ASCs. External radioactive markers were placed at the second cervical (C2) vertebra, the first (T1), seventh (T7), thirteenth (T13), and eighteenth (T18) thoracic vertebrae, as well as the third sacral vertebra (S3). After AO injection, the radioactive signal can be observed all the way to the cranial lumbar spine (**a–f**), whereas no cranial migration of radiolabeled ASCs can be observed after lumbosacral injection (**g–i**)

Immediately after AO injection of radiolabeled ASCs, a radioactive signal was identified with the maximal intensity at the site of injection and extending cranially into the caudal aspect of the cranial vault and caudally to the level of the second cervical vertebra (Fig. 1a, c). One hour after injection, a radioactive signal was present at the level of the first thoracic vertebra and, at 5 h, the radioactive signal could be identified reaching the lumbar spine (Fig. 2). At 24 h postinjection, the radioactive signal was still present and strong in the cranial cervical vertebral canal, with a weaker signal seen within the lumbar spine. The radioactive signal remained the most intense at the injection site at all time points.

After LS injection, a radioactive signal was immediately visible at the level of the last lumbar vertebra and cranial sacrum (Fig. 1b, d). Over time, the signal extended caudally to the posterior dural sac, while a minimal radioactive signal extended cranially (Fig. 2). The signal was still identified at 24 h postinjection.

**Fig. 3:**
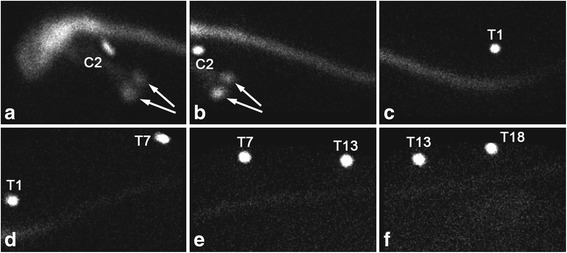
Scintigraphic images obtained 5 h after AO injection of free radiolabel. External radioactive markers were placed at the second cervical (C2) vertebra (**a**, **b**), the first (T1) (**c**, **d**), seventh (T7) (**d**, **e**), thirteenth (T13) (**e**, **f**), and eighteenth (T18) (**f**) thoracic vertebrae. A weak radioactive signal from free ^99m^Tc-HMPAO reached the lumbar area by 5 h after injection (**f**). Uptake was apparent in the thyroid gland (**a**, **b**) due to diffusion of the free label outside of the subarachnoid space (arrows)

The distribution and progression of the free ^99m^Tc-HMPAO radioactive signal after AO administration was similar to the injection of labeled ASCs, with the signal reaching the cranial thoracic area after 1 h and the sacral area after 5 h (Fig. 3). Mild uptake was apparent in the thyroid gland after the control injection (Fig. 3a, b). This was not seen with the labeled ASC injection.

Radiolabel persistence at the injection site was quantified (Table 2). Persistence decreased over time with all three injections. The persistence after AO injection at the later time points was lower than after LS injection. The persistence after the control injection was lower at all time points except for that at 24 h.

**Table 2:** Quantification of radiolabel persistence (%) at the injection site over time

Time	AO	LS	AO-C
0	100%	100%	100%
30 min	93%	87%	79%
1 h	80%	85%	58%
5 h	37%	48%	32%
24 h	13%	18%	21%

*AO*, atlanto-occipital, *AO-C*, atlanto-occipital control, *LS*, lumbosacral

#### Horses did not develop anti-ASC antibodies after intrathecal administration

No horses (0/4) developed detectable anti-ASC alloantibodies. Percent specific IgG binding ranged from 0 to 2.8% at all time points with no change from baseline (time 0), 30 days, or 14 months following Ad-MSC administration in any of the horses.

#### Horses did not develop anti-BSA antibodies after intrathecal administration

Three of the four horses had high titers to BSA at day 0 and those titers remained high at day 30 and at 14 months following Ad-MSC administration. One horse had a low positive titer to BSA at day 0 and that titer remained low at day 30 following Ad-MSC administration. This horse converted to a high BSA titer at the 14 month recheck, likely due to recent vaccination.

### ASC injection into neurologically diseased horses

**Fig. 4:**
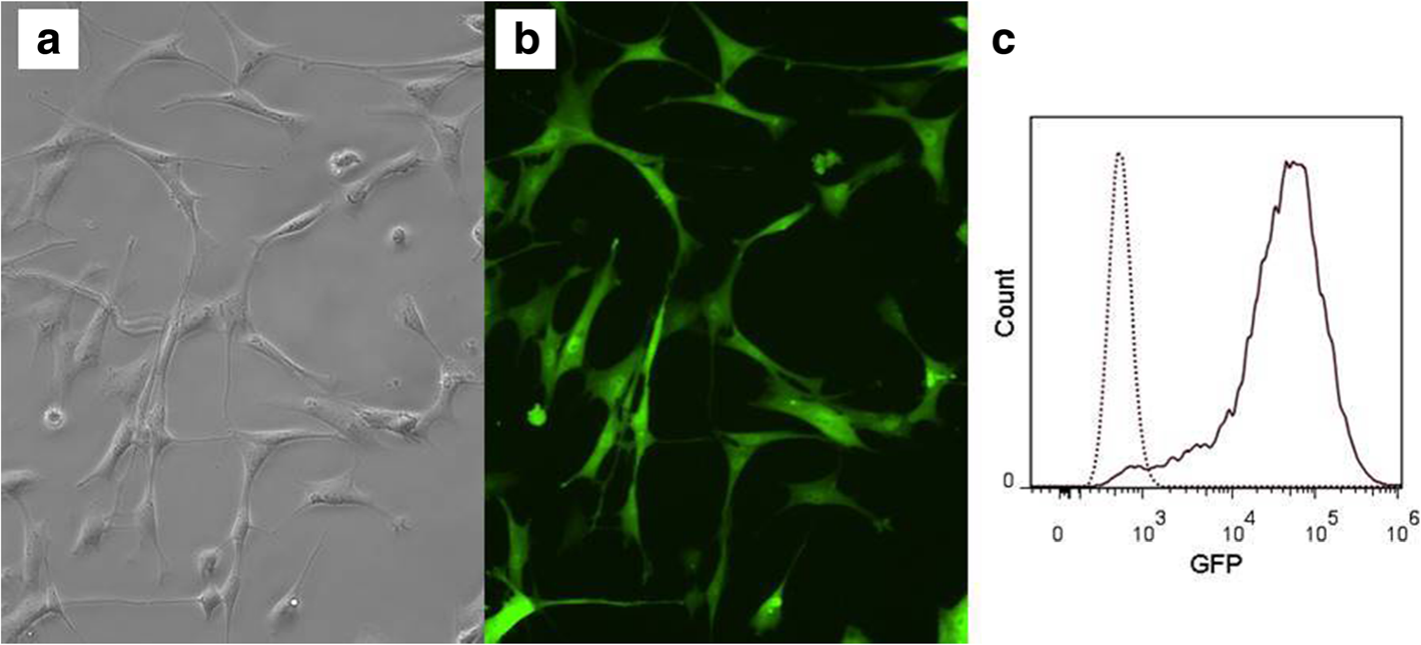
GFP-labeled ASCs before injection into diseased horses. **a** ASCs without green fluorescent protein (GFP). (**b**) GFP-labeled cells. **c** Flow cytometric histogram image of GFP-labeled ASCs

ASCs labeled with GFP (Fig. 4) were administered via AO injection with no adverse effects. The complete blood count and biochemical profile showed no alterations prior to or after ASC injection. CSF analysis showed a mild increase in protein concentration before ASC injection in one horse.

The neurological status of the diseased horses remained unchanged throughout the different time points of examination following the administration of ASCs. The C1–C6 myelopathy was characterized by ataxia, tetraparesis, hypermetric gait, and proprioceptive deficits of all limbs. The neurological deficits were symmetrical and graded 3 of 5.

### Post-mortem examination findings

All diseased horses were subject to a complete post-mortem examination that showed lesions consistent with equine CVCM. Chiefly, spinal cords from affected animals exhibited swollen myelin sheaths, axonal swelling and spheroid formation, digestion chamber formation, and multifocal glial nodules. Affected spinal cord segments ranged in severity but were typically most severely affected in C4 to C6 (Additional file 2). Spinal cord segments showed no GFP-labeled ASCs at the site of the lesions and no immunolabeling towards GFP was detected in any patients (data not shown).

## Discussion

Neurological diseases of horses are important causes of ataxia, weakness, and decreased performance. The pathophysiology of various neurologic diseases can be complex, multifactorial, and/or poorly understood. Depending on etiology, certain neurologic conditions can represent a therapeutic challenge, especially those that result in profound brain and spinal cord injury [20–22, 33, 43]. To overcome the limited capacity of CNS regeneration, a possible alternative would be to use MSCs to assist in modulating neuroinflammation and neuroregeneration.

Data from this exploratory study suggest that the intrathecal administration of relatively high doses of allogeneic, culture-expanded ASCs is well tolerated in healthy horses regardless of whether the cells are administered at the AO or LS space. In addition, ASCs after AO injection appear to distribute more efficiently through the subarachnoid space. Another study in horses also showed the safety of intrathecal transplantation through the AO space of bone marrow-derived MSCs (BM-MSCs) with a much lower cell dose (1 × 10^6^ BM-MSCs) [28]. Other human studies have demonstrated the safety of intrathecal injection of autologous MSCs [6, 27, 28, 44] with a few studies using allogeneic MSCs [45, 46].

A single intrathecal administration of allogenic ASCs to healthy horses did not elicit an anti-MSC alloantibody response. These findings may suggest that, in the absence of disease and breakdown of the blood–brain barrier, a systemic response to allogenic cells will not be mounted. In our previous study, low-dose cell administrations or a single dose also did not elicit an anti-MSC antibody response whereas multiple high doses into a tendon lesion did elicit a response [41, 47]. The percent of background binding of equine IgG to equine Ad-MSCs was similar in this study (< 2.8%) to what we noted previously (background IgG binding varied from 2.4–7.5%). These findings suggest that the assay is fairly robust and repeatable.

In agreement with our previous study, the majority of horses (89%) were positive for anti-BSA antibodies prior to and after MSC injection. Anti-BSA antibodies develop in horses due to frequent vaccination with products created in FBS-containing media [41]. Similar to our previous study, anti-BSA titers did not increase in the horses in this study.

There is no consensus on the best route to deliver MSCs to the neurological system [48]. Studies have variably used intravenous [14], intrathecal [2], intranasal [49], direct lesional, and intracerebral [50] injections. Theoretically, intrathecal injection could be more effective since relatively large MSCs would not have to pass the blood–brain barrier. In support of this, several studies have reported beneficial effects in mice and humans after intrathecal MSC administration for CNS diseases [10, 18, 51–54]. However, at least one serious adverse event (acute disseminated encephalomyelitis) has been reported after intrathecal BM-MSC administration in a human [55].

In this study, both AO and LS transplantation of ASCs was technically easy and safe to perform, with no demonstrated adverse effects in horses. Furthermore, the neurologic status was not altered in any of the horses (healthy or diseased) in this study. The caudal distribution of the labeled ASCs after AO injection and the lack of cranial migration after LS injection suggest that the cells are progressing in the direction of CSF flow in the absence of any identified spinal cord lesions. The lower persistence at the AO injection site when compared to LS is consistent with the subjective assessment of migration of ASCs. The caudal migration of ASCs injected at the AO space is likely responsible for this lower persistence.

Based on our study, atlanto-occipital centesis is the preferred route of administration to facilitate getting the highest concentration of MSCs to the cervical area. Given that both routes are safe, determining the best site for ASC transplantation should take into account the safety of the horse (for example, general anesthesia) and lesion location [56]. Lumbosacral puncture is a relative easy procedure that does not require general anesthesia [57]. This approach may be preferable in animals showing neurological signs localized caudal to T3 spinal cord segments. Although AO puncture is routinely performed under general anesthesia [58], it has been reported that it could be performed in the standing horse with appropriate sedation [59]. However, puncture of the spinal cord or brainstem is a potential risk. Another technique that has been described in the standing horse is the collection of CSF between the atlas and axis [30].

The relatively low persistence at 24 h also observed with the LS injection despite the lack of demonstrated local migration is similar to the persistence that has been reported with local intralesional injection of MSCs in the equine superficial digital flexor tendon (24% at 24 h) [60]. This might be due to systemic distribution of the MSCs after injection but this might also reflect a limitation of the tracking technique. Suboptimal label persistence has been demonstrated after injection of ^99m^Tc-HMPAO-labeled MSCs in dogs [38]. Although it is harder to demonstrate in horses due to physical limitations in imaging the whole body (size and signal attenuation), it is likely that the measured persistence is an underestimation of the actual cell persistence due to loss of label from some of the cells.

The concern about the presence of the free label was the justification for performing a control injection. The similar distribution of the labeled ASCs and the free label suggest that both are distributing following the CSF flow which is mainly unidirectional from cranial to caudal [61]. The lower initial persistence of the free label can be explained by a faster distribution or higher systemic absorption that can be explained by the smaller size of the label compared with the ASCs. The higher persistence at 24 h with free label might be explained by fixation of some of the label in local cells. The uptake in the thyroid region observed with the control injection confirms systemic distribution of the free label and absorption of free technetium by the thyroid gland.

We were unable to find GFP-labeled ASCs transplanted into diseased horses 7 or 15 days after ASC injection. This finding could be due to the fact that: 1) the neurologic lesions were chronic and did not attract ASCs to the site; 2) ASCs were not restricted to the spinal cord area and they could have migrated outside the CSF or distributed throughout the subarachnoid space; or 3) ASCs could have undergone cell death.

A study with ASCs labeled with Qtracker 655 quantom dots showed that ASCs were still found at an equine tendon lesion site 1-week postinjection [62]. Another study compared administration of GFP-labeled ASC trough intravenous, intraperitoneal, and subcutaneous routes in mice, and after 75 days eGFP-bright cells were found in the brain, heart, liver, lung, kidney, and omental fat by polymerase chain reaction (PCR) and cytospin analyses [39]. It has also been demonstrated in mice that MSCs injected intra-arterially selectively engraft in the bone marrow after a localized radiation up to 33 weeks. In this study, the authors used bioluminescent imaging to measure cell distribution of monomeric red fluorescent protein/luciferase MSCs [63].

Some studies have reported MSC apoptosis after in vivo injection [64–67], but despite this it was shown to be crucial for the MSC immunosuppression function [65] and to improve cardiac function through secretion of the anti-inflammatory factor tumor necrosis factor (TNF)-α-induced protein 6 (TNAIP6 or TSG-6) [67].

Limitations of the study include the small number of horses, making interpretation of quantitative data difficult (for example, the increase in CSF protein in one horse). There were small changes in the CSF nucleated cell count both prior to and after ASC injection in four out of five horses. These findings might represent normal variation or mild subclinical disease, although all animals were neurologically normal on examination.

## Conclusions

Our results demonstrate that AO and LS intrathecal injection of allogeneic ASCs is safe and easy to perform in horses. Additionally, due to the flow of CSF from cranial to caudal, AO administration of ASCs had a better distribution within the subarachnoid space and presumably to the spinal cord from the cervical to the lumbosacral region, suggesting that this approach might be more suitable for cranial lesions in the spinal cord. ASCs could not be found at 15 days after injection at the site of injury in horses with CVCM, suggesting that ASCs did not have time to reach the lesion site or that ASCs did not stay/survive in the spinal cord for this period of time in a high enough number to be detected. Additional work needs to be performed to determine if multiple intrathecal allogeneic MSC injections are well tolerated as well as the efficacy of MSCs to treat horses with inflammatory or degenerative lesions of the nervous system.

## Additional files

Additional file 1: Representative image of equine ASC phenotype. Equine ASC phenotype panel. Positive markers: CD44, CD29, CD90, and MHC I. Negative markers: F6B and MHC II. (JPEG 46 kb)

Additional file 2: Representative images of neurologic patients, spinal cord cross-sections, H&E stain. A) Several dilated myelin sheathes are evident that B) contain macrophages consistent with digestion chambers (arrows). C) Swollen axons are present with spheroid formation (*). D) Multifocal glial nodules (arrowhead) are present within the white matter. Scale bar = 100 μm. (JPEG 92 kb)

## Abbreviations

^99m^Tc-HMPAO: ^99m^Technetium-hexamethyl-propylene-amine-oxyme
AO: Atlanto-occipital
ASC: Adipose-derived mesenchymal stem cell
BM-MSC: Bone marrow-derived mesenchymal stem cell
BSA: Bovine serum albumin
CEH: Center of Equine Health
CNS: Central nervous system
CSF: Cerebrospinal fluid
CVCM: Cervical vertebral compressive myelopathy
DAPI: 4,6-Diamidino-2-phenylindole
ELISA: Enzyme-linked immunosorbent assay
EPM: Equine protozoal myeloencephalitis
FBS: Fetal bovine serum
GFP: Green fluorescent protein
H&E: Hematoxylin and eosin
IACUC: Institutional Animal Care and Use Committee
IFAT: Immunofluorescent antibody test
LS: Lumbosacral
MSC: Mesenchymal stem cell
PFA: Paraformaldehyde
RML: Regenerative Medicine Laboratory
ROI: Region of interest
VMTH: Veterinary Medical Teaching Hospital
