# Supplementary figures and images for: Safety and tracking of intrathecal allogeneic mesenchymal stem cell transplantation in healthy and diseased horses
Source: Stem Cell Res Ther. 2018 Apr 10;9:96. doi: 10.1186/s13287-018-0849-6 (PMC5891950; doi:10.1186/s13287-018-0849-6)

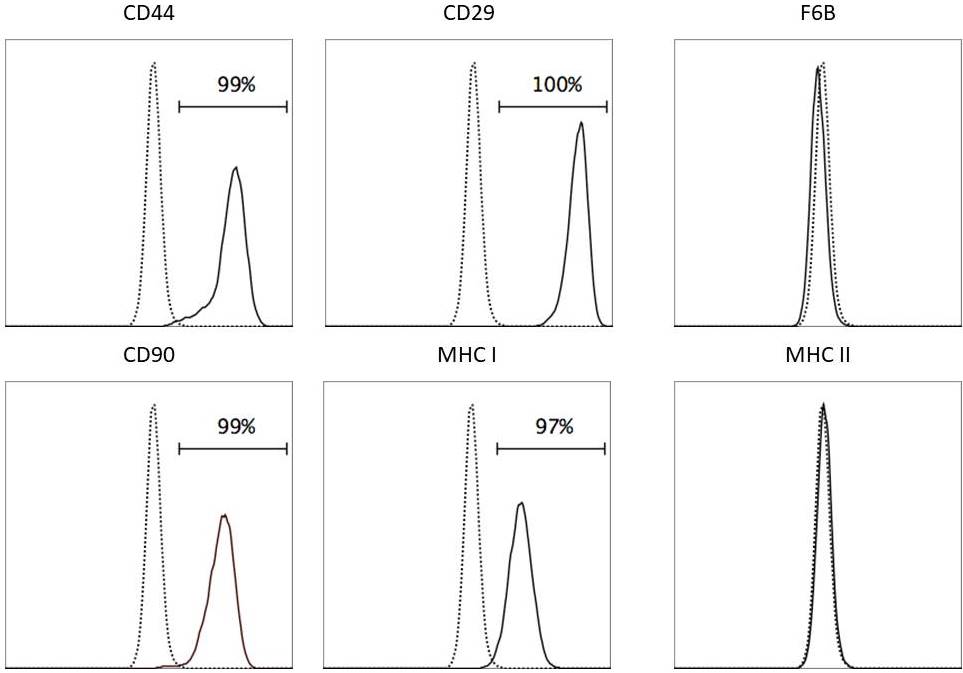

Supplement: Supplementary file 1 — Representative image of equine ASC phenotype. Equine ASC phenotype panel. Positive markers: CD44, CD29, CD90, and MHC I. Negative markers: F6B and MHC II. (JPEG 46 kb) [file 13287_2018_849_MOESM1_ESM.jpg]

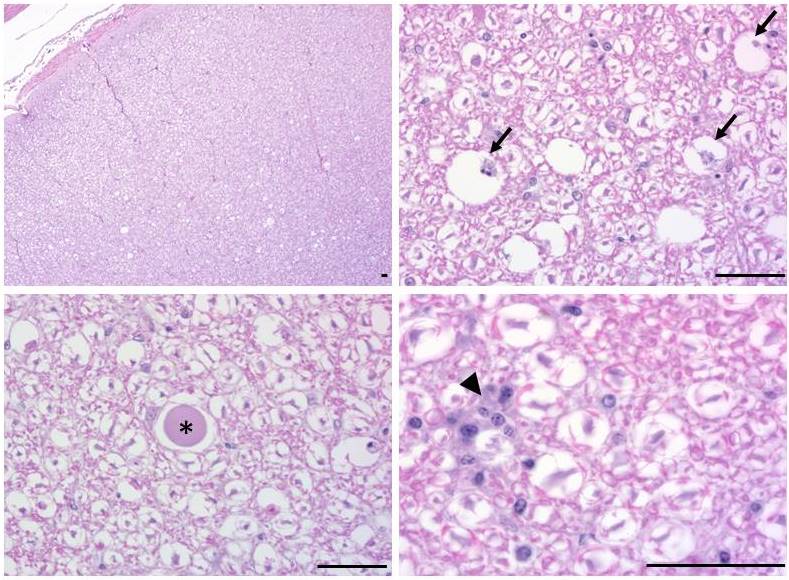

Supplement: Supplementary file 2 — Representative images of neurologic patients, spinal cord cross-sections, H&E stain. A) Several dilated myelin sheathes are evident that B) contain macrophages consistent with digestion chambers (arrows). C) Swollen axons are present with spheroid formation (*). D) Multifocal glial nodules (arrowhead) are present within the white matter. Scale bar = 100 μm. (JPEG 92 kb) [file 13287_2018_849_MOESM2_ESM.jpg]
